# Supplementary material for: The interplay at the replisome mitigates the impact of oxidative damage on the genetic integrity of hyperthermophilic Archaea
Source: eLife. 2019 Jun 11;8:e45320. doi: 10.7554/eLife.45320 (PMC6559790; doi:10.7554/eLife.45320)
Supplement: Supplementary file 1. — The location of the fluorescent labels is indicated by *. [file elife-45320-supp1.doc]

| p/t | Sequence (X=dG/8-oxodG) (Y=dA/dC/dT) | 5' Label |
| --- | --- | --- |
| p/t-1 | *5'TGCCAAGCTTGCATGCC | Cy5 |
|  | 3'ACGGTTCGAACGTACGGACGTCCAGCTGAGATXTCCTAGGGGCCCATGGCTCGAGCTTAAGCATTAGTACCAGTATCGACAAAGGAC | - |
| p/t-2 | *5'TGCCAAGCTTGCATGCC 5'AGGTCGACTCTACAGGATCCCCGGGTACCGAGCTCGAATTCGTAATCATGGTCATAGCTGTTTCCTG | Cy5 |
|  | 3'ACGGTTCGAACGTACGGACGTCCAGCTGAGATXTCCTAGGGGCCCATGGCTCGAGCTTAAGCATTAGTACCAGTATCGACAAAGGAC | - |
| p/t-3 | *5'TGCCAAGCTTGCATGCCTGCAGGTCGACTCTA | Cy5 |
|  | 3'ACGGTTCGAACGTACGGACGTCCAGCTGAGATXTCCTAGGGGCCCATGGCTCGAGCTTAAGCATTAGTACCAGTATCGACAAAGGAC | - |
| p/t-4 | *5'TGCCAAGCTTGCATGCCTGCAGGTCGACTCTAY | Cy5 |
|  | 3'ACGGTTCGAACGTACGGACGTCCAGCTGAGATXTCCTAGGGGCCCATGGCTCGAGCTTAAGCATTAGTACCAGTATCGACAAAGGAC | - |
| p/t-5 | *5'TGCCAAGCTTGCATGCCTGCAGGTCG | Cy5 |
|  | 3'ACGGTTCGAACGTACGGACGTCCAGCXTCCTAGG |  |
| p/t-6 | *5'TGCCAAGCTTGCATGCC | Cy5 |
|  | 3'ACGGTTCGAACGTACGGACGTCCAGCXTCCTAGG* | FAM |

Supplementary File 1
